# LC-ESI-MS/MS Analysis and Pharmacokinetics of GP205, an Innovative Potent Macrocyclic Inhibitor of Hepatitis C Virus NS3/4A Protease in Rats

**DOI:** 10.3390/molecules20034319

**Published:** 2015-03-06

**Authors:** Nan Yang, Qiushi Sun, Zihua Xu, Xiuyun Wang, Xin Zhao, Yuqing Cao, Li Chen, Guorong Fan

**Affiliations:** 1 School of Pharmacy, Second Military Medical University, Shanghai 200433, China; E-Mails: luckyyangnan123@163.com (N.Y.); sunnyautumn.cool@163.com (Q.S.); Xuzihua-668585@163.com (Z.X.); chunyuhan2012@163.com (X.W.); yoyo0132@163.com (X.Z.); caoyuqing1109@163.com (Y.C.); 2 Shanghai Key Laboratory for Pharmaceutical Metabolite Research, Shanghai 200433, China; 3 Fujian University of Traditional Chinese Medicine, Fuzhou 350108, China; 4 Ginkgo Pharma Co. Ltd., Suzhou 205125, China; E-Mail: chenli@ginkgopharma.com

**Keywords:** GP205, LC-ESI-MS/MS, pharmacokinetics, HS3/4A, HCV

## Abstract

A high-throughput, sensitive and specific LC-ESI-MS/MS method was established for the quantitative determination of GP205, a potent inhibitor of hepatitis C virus NS3/4A protease, in rat. The analyte was isolated from 25 μL plasma sample by 96-well LLE. Good linearity was achieved within the concentration range of 2–5000 ng/mL (*r*^2^ > 0.996). The intra- and inter-day precision was less than 10%. The accuracy ranged from 0.8% to 5.5% for GP205 in quality control samples at three levels. GP205 was stable during the analysis and the storage period. The method was successfully applied to pharmacokinetic studies of GP205 in Sprague-Dawley rats. The pharmacokinetic profiles of GP205 at three dose levels with oral administration and one dose level with intravenous administration were successfully studied for the first time in SD rats, respectively. After single oral administration of GP205 at the doses of 2.5, 5, 10 mg/kg, respectively, *C*_max_ and AUC_0-τ_ were proportional to the doses given. The absolute bioavailability was estimated as 34% based on the AUCs of oral administration at the dose of 5 mg/kg and intravenous administration at the dose of 1 mg/kg. The data presented in this study provides useful information for further study for GP205.

## 1. Introduction

Hepatitis C virus (HCV) is a chronic infection that affects an estimated 170~200 million people worldwide, or 3% of the global population. Every year, there are about 3 to 4 million new hepatitis C-infected patients in developing and transitional economy countries [1,2]. HCV is a positive-strand RNA virus belonging to the family Flaviviridae [3,4]. The symptoms of HCV infection are not obvious in the early stages of the disease, however, as the disease progresses about 50%~80% of HCV patients may develop serious liver disease, including cirrhosis and hepatocellular carcinoma. In some cases, hepatitis C can remain asymptomatic, even after significant liver damage has occurred [5,6,7]. Ever since the identification of this virus, many researchers have been working on effective drugs to improve the treatment of hepatitis C. The standard of care for the treatment of HCV infection for the past decade has consisted of pegylated interferon-α (peg-IFN-α) in combination of ribavirin (RBV). This combination therapy could increase the sustained virologic response (SVR) rates to ~50% in patients with HCV genotypes 1 and 4, and ~80% in patients with HCV genotypes 2 and 3. Response is genotype dependent [8,9,10]. This therapy is difficult to tolerate, coupled with many toxicities and serious side effects, which makes patients discontinue their treatment [11,12,13]. Therefore, there is intense interest in the development of more effective HCV replication inhibitors with less side effects.

Efforts to improve HCV treatment include the development of direct acting antivirals which target specific pathways interfering with HCV infection and replication. Multiple viral proteins essential for replication have been characterized [14,15]. A clinical proof of concept has been demonstrated for small-molecule inhibitors that target structural and nonstructural proteins, including NS3/4A protease [16,17], NS5B polymerase (both active site and allosteric inhibitors) [18,19,20], NS4A [21], and recently, NS5A [22]. NS3 protease has demonstrated a vital role in the replication of the HCV virus. It is a pivotal enzyme required for maturation of HCV and assists in the processing of the HCV polyprotein by cleaving four downstream sites [23,24,25,26,27,28,29,30,31,32].

In recent years, several NS3/4A protease inhibitors have moved into human clinical trials. In short-term clinical studies, inhibitors of HCV NS3/4A protease [16,17,23,33,34] have demonstrated marked antiviral activity and lowered (by several orders of magnitude) plasma HCV RNA, which is the principal biomarker of HCV replication *in vivo* [35,36,37,38,39]. The first generation clinical proof-of-concept for an HCV direct active antiviral inhibitor had been shown for BILN-2061, which was a rapidly reversible, P1-P3-constrained macrocyclic compound with good activity of antiviral *in vitro* and high absorption *in vivo* [27,35]. However, further development was halted because of cardiotoxocity identified in rhesus monkeys [40]. Then the second generation of keto-amide HCV NS3/4A protease inhibitors was developed, for example VX-950 (telaprevir) [36,37] and SCH-503034 (boceprevir) [38], which covalently bind to the active-site serine of the protease in a slowly reversible manner. In May 2011, boceprevir (Victrelis^TM^, Merck, Rahway, NJ, USA) and telaprevir (Incivek^TM^, Vertex Pharmaceuticals, Boston, MA, USA), a new class of medicines known as NS3/NS4A protease inhibitors were approved by the FDA for use in HCV genotypes 1-infected patients [41], and the standard of care for the treatment of chronic HCV was changed to triple therapy with peg-IFN-α, RBV, and an HCV protease inhibitor. The combination regimens can significantly improve treatment responses, but the side effect profile of this particular compound are more common and more severe than with peg-IFN-α and RBV alone, which might compromise its clinical effectiveness [42,43]. More recently, the development of the third generation HCV NS3/4A protease inhibitors has turned to macrocyclic compounds to find more efficient inhibitors with longer half-lives. A number of macrocyclic HCV NS3/4A protease inhibitors structurally related to BILN-2061, including ITMN-191 [44], TMC435350 [45] and BI201335 [46], have been evaluated in early clinical stages. Among these, MK-1220 containing a P2 to P4 macrocycle (in contrast to the P1 to P3 linker present in BILN-2061 and several other known inhibitors) has been reported to limit the improved preclinical pharmacokinetics [47,48,49]. Optimization of the P2 heterocycle substitution pattern as well as the P3 amino acid led to analogs with greatly improved plasma exposure and excellent enzyme potency and cellular activity following oral dosing in both rats and dogs [50].

**Figure 1:**
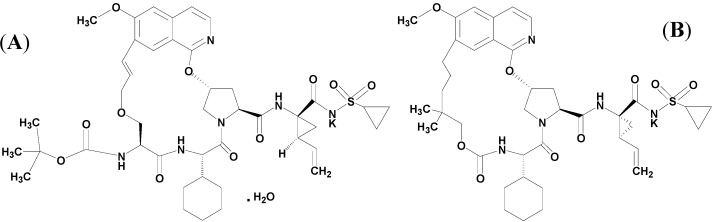
Chemical structures of GP205 (**A**) and MK-1220 (**B**).

In China, during 2010, it was estimated that approximately 30 million people, or about 2.1% of China’s population had been infected with HCV (the largest number of HCV infected people of the countries in the world) [51]. The statistical data showed that at least four genotypes (1, 2, 3 and 6) had been found and the major prevalent subtype was 1b, that accounts for about 73% of all HCV infections [52,53]. To solve this urgent problem, an oral drug candidate named GP205 (Figure 1A) has been discovered in China [54,55]. GP205 is a potent macrocyclic inhibitor of HCV NS3/4A protease, which is a structural modification of MK-1220 (Figure 1B) and is highly efficient in the interference with the normal catalytic functions of NS3/4A, and blocking the replication of HCV.

*In vitro* experiments have revealed that GP205 could significantly inhibit the catalytic activity of the NS3 enzyme which is the catalyst in genotype 1b HCV. The GP205 IC_50_ value is 4.0 nM while that of the positive drug telaprevir is 95 nM and for MK-1220 it is 11 nM. For an oral administration drug, anti-HCV activity tests in cell culture are not enough to estimate the exposure level *in vivo*. But the lack of a robust small-animal model for HCV infection has generally forced scientists to rely on animal pharmacokinetics as surrogate indicators of efficacy before clinical trials. To investigate the absorption characteristics and preclinical pharmacokinetics study of GP205 *in vivo*, we established a high-throughput, sensitive, specific liquid chromatography-tandem mass spectrometry (LC-MS/MS) method to measure the concentration of GP205 in Sprague Dawley (SD) rat plasma. Following 96-well liquid-liquid extraction (LLE), 25 μL of plasma sample was separated and monitored by an LC-MS/MS system in an assay requiring only 4 min per sample. Results of the full validation presented here demonstrate that the method is suitable for analyzing GP205 in SD rat plasma. Based on this method, a pharmacokinetic study of GP205 at three dose levels with oral administration and one dose level with intravenous administration was successfully performed for the first time in SD rats.

## 2. Results and Discussion

### 2.1. Method Development

#### 2.1.1. Sample Preparation

In sample preparations, considering the complex procedures and high cost of solid phase extraction (SPE) in the 96-well plate format, we just compared protein precipitation (PPT) with LLE to choose the better pretreatment method for GP205 and the internal standard (IS). Our initial sample preparation was based on PPT with methanol or acetonitrile because of its simplicity and universality for drug molecules in plasma. Strong sample matrix interference and low recoveries of GP205 and IS was presented by PPT, while LLE gave sufficient purification with high recoveries and was adopted in the end. Ethyl acetate and methyl *tert*-butyl ether were tested, and finally methyl *tert*-butyl ether was chosen because of its high extraction efficiency, less interference and good peak shape.

#### 2.1.2. LC-MS/MS Optimization

**Figure 2:**
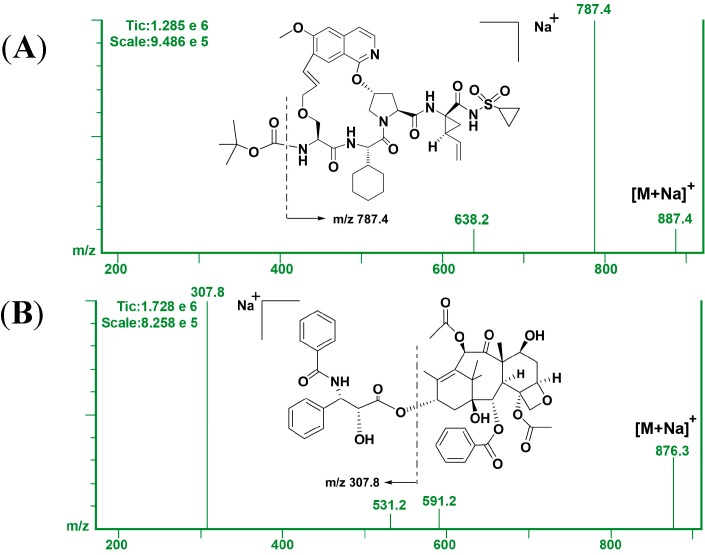
Representative product ion mass spectra of (**A**) GP205 and (**B**) paclitaxel (IS).

To optimize the MS conditions, the positive and negative mode were both tested. Multiple reaction monitoring (MRM) conditions were established for GP205 and paclitaxel (IS) by mixing 1 μg/mL of each compound (20 μL/min) with mobile phase (200 μL/min) and infusing the mixture via a tee-union into the mass spectrometer. In the positive mode the response of the analytes was more than 100 times higher than that in negative mode. The analyte and IS formed adduct ions of *m*/*z* 887.5 [M+Na]^+^ and 876.3 [M+Na]^+^, respectively. In the product ion spectra, several fragment ions were obtained, and then the ions at *m/z* 787.4 and 307.8 were chosen in the acquisition of GP205 and IS, respectively for their much greater intensity. Representative product ion mass spectra of GP205 and IS are shown in Figure 2. The chromatographic conditions were optimized through several trials to achieve good resolution and symmetric peak shapes for analyte and IS, as well as a short analysis time. Modifiers, such as ammonium acetate and formic acid alone or in combination in different concentrations were added. Their percentage was optimized to maintain the peak shape while being consistent with good ionization and fragmentation in the mass spectrometer. It was found that a mixture of methanol-water-formic acid (95:5:0.1, v/v/v) could achieve theses purpose and that mixture was finally adopted as the mobile phase. According to the structure and polarity of GP205, a Zorbax SB-C18 column (100 mm × 3.0 mm, 3.5 μm) was selected with a flow rate of 0.3 mL/min to achieve an efficient chromatographic separation between the analytes and the endogenous plasma components for eliminating the matrix effects.

### 2.2. Method Validation

#### 2.2.1. Selectivity

**Figure 3:**
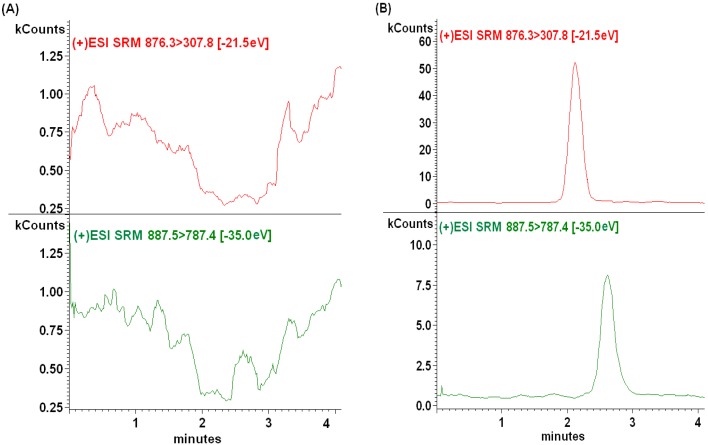

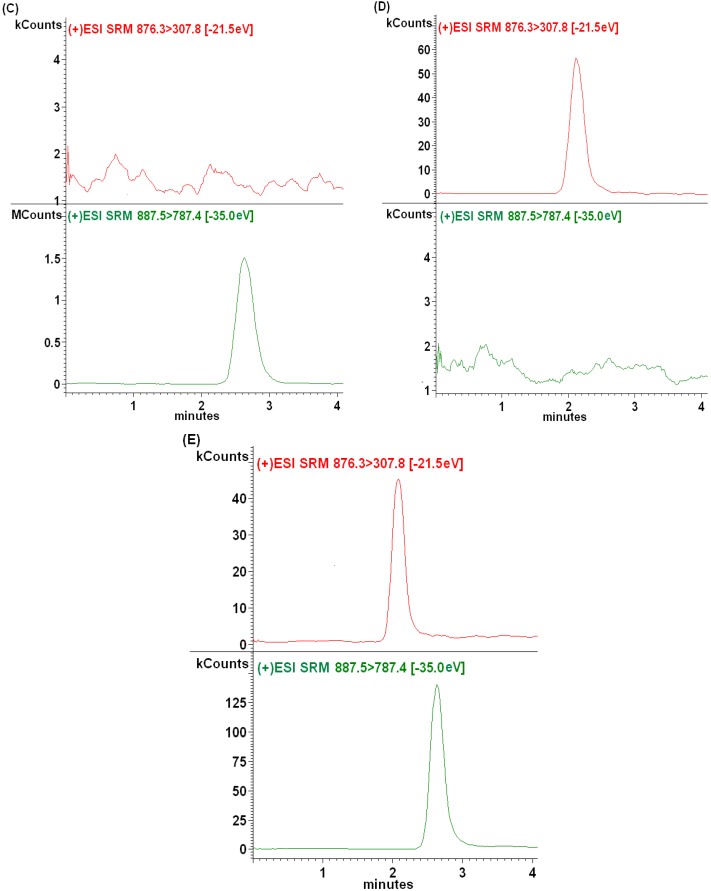
Representative MRM chromatograms of GP205 and IS in SD rat plasma samples. (**A**) A blank plasma sample; (**B**) a blank plasma sample spiked with GP205 (2 ng/mL) and IS (100 ng/mL); (**C**) blank plasma spiked with GP205 only (5000 ng/mL); (**D**) blank plasma spiked with IS only (100 ng/mL); (**E**) a plasma sample from a rat 1 h after oral administration of GP205 at the dose of 2.5 mg/kg. The assayed concentration of GP205 in this sample was 716.39 ng/mL.

The selectivity of the method was tested by comparing the chromatograms of blank plasma sample and the spiked plasma sample. Six blank samples from six lots of rat plasma were processed with and without the standards. Under the described chromatographic conditions, a good separation was achieved and no obvious endogenous interferences from rat plasma were observed. The retention times of GP205 and IS were 2.5 and 2.1 min, respectively. Typical chromatograms of GP205 and IS are presented in Figure 3A.

In addition, the “cross-talk” between the two channels in the LC-MS/MS system was evaluated. Figure 3C,D clearly showed no MS/MS response from the analyte into the IS channel and *vice versa*.

#### 2.2.2. Sensitivity and Linearity

The lower limit of quantitation (LLOQ) was 2 ng/mL for GP205. The reproducibility of LLOQ was determined by examining five LLOQ samples independent from the standard curve, and the accuracy and precision was 5.2% and 8.0%, respectively. Calibration curves were constructed by plotting the peak area ratios (analyte/IS) of plasma standards *vs.* nominal concentrations. The best linear fit and least-squares residuals for the calibration curve were achieved with a 1/x weighing factor, giving a mean linear regression equation for the calibration curve of y = 0.0078x + 0.0127, *r*^2^ = 0.9984, where y represents the peak area ratios of GP205 to that of IS, and x represents the plasma concentration of GP205 in ng/mL. Calibration curves of four different lots of plasma were linear in the range of 2–5000 ng/mL for GP205 with *r*^2^ ≥ 0.996. The difference between the nominal standard concentration and the back-calculated concentration from the weighed linear regression line was varied from −10% to 15% for each point on the standard curve. The upper limit of quantitation (ULOQ) of the assay, defined as the highest concentration on the standard curve was 5000 ng/mL for GP205. The higher concentrations were investigated but the linearity was not good. As a result, the actual concentration above the ULOQ will be diluted with blank rat plasma and analyzed.

#### 2.2.3. Accuracy and Precision

The intra- and inter-day precision and accuracy were estimated by analyzing five replicates at three quality control (QC) concentrations on the same day and on three consecutive days. The precision was determined by calculating the relative standard deviation (RSD) for intra- and inter-day replicates. The accuracy of the method was determined by calculating the percentage deviation observed in the analysis of QCs and expressed as the relative error (RE). The method showed good accuracy and precision. Table 1 shows a summary of intra- and inter-day accuracy and precision for GP205 from QC samples, respectively. In this assay, the intra-day precision was less than 10% for each QC level of GP205 and the inter-day precision was less than 5%. The accuracy ranged from 0.8% to 5.5%.

**Table 1:** Summary of accuracy and precision for the determination of GP205 in SD rat plasma.

Nominal Concentration (ng/mL)	Intra-Day (*n* = 5)			Inter-Day (*n* = 15)		
	Measured Concentration (ng/mL) (Mean ± S.D.)	RSD (%)	RE (%)	Measured Concentration (ng/mL) (Mean ± S.D.)	RSD (%)	RE (%)
5	5.07 ± 0.36	7.1	0.8	5.18 ± 0.26	5.0	3.0
100	106.15 ± 2.99	2.9	5.5	103.53 ± 5.16	5.0	2.9
4000	4062.09 ± 93.81	2.4	1.0	4059.29 ± 110.69	2.8	0.9

#### 2.2.4. Extraction Recovery and Matrix Effect

The extraction recovery and the matrix effect on LC-MS/MS detection were evaluated for analyte and the IS. To investigate extraction recovery, a set of QC samples (*n* = 5 at each concentration in the unique lots of plasma) were prepared by spiking GP205 into plasma at 5, 100, 4000 ng/mL, and IS at about 100 ng/mL. The samples were subsequently processed using the procedure described previously. A second set of blank plasma samples were extracted and spiked the analyte and IS to obtain the corresponding concentrations with the set 1. Extraction recovery values for analyte and IS were determined by calculating the ratios of the raw peak areas of the QC samples to that of the corresponding standard solution spiked in extracted blank plasma.

The matrix effects are generally due to the influence of coeluting compounds on the actual analyte ionization process. The effects of the plasma matrix on ionization efficiency were expressed as the ratio of the mean peak area of analyte spiked after extraction from five different lots of plasma (*i.e.*, lots originating from five SD rats, respectively) to that of the neat standards at corresponding concentrations. The results obtained were within the acceptable limit, which indicated that there was no significant lot-to-lot variation in matrix effects. The recovery and matrix effect results are indicated in Table 2.

**Table 2:** Extraction recovery and matrix effect of GP205 in SD rat plasma (*n* = 5).

Nominal Concentration (ng/mL)	Peak Area ^a^ (e^5^) (A) (Mean ± S.D.)	Peak Area ^b^ (e^5^) (B) (Mean ± S.D.)	Peak area ^c^ (e^5^) (C) (Mean ± S.D.)	Extraction Recovery ^d^ (%) (A/B)	Matrix Effect ^e^ (%) (B/C)
5	0.39 ± 0.02	0.45 ± 0.02	0.47 ± 0.02	86.7	95.7
100	5.20 ± 0.11	5.91 ± 0.19	6.09 ± 0.16	88.0	97.0
4000	192.50 ± 12.01	206.90 ± 5.62	215.47 ± 5.44	93.0	96.0
100 (I.S.)	7.27 ± 0.47	7.83 ± 0.46	8.45 ± 0.22	92.9	92.7

^a^ Standards spiked before extraction. ^b^ Standards spiked after extraction. ^c^ Neat standards. ^d^ Extract recovery (%) expressed as the ratio of the mean peak area of the analytes spiked into plasma pre-extraction (A) to the mean peak area of the analytes spiked into plasma post-extraction (B). ^e^ Matrix effect expressed as the ratio of the mean peak area of the analytes spiked into plasma post-extraction (B) to the mean peak area of the neat standards (C).

The mean extraction recoveries of GP205 at concentrations 5, 100 and 4000 ng/mL were 86.7%, 88.0% and 93.0%, respectively, and the extraction recovery of the IS was 92.85%. The mean matrix effect values obtained were 95.7%, 97.0% and 96.0% for GP205 at 5, 100 and 4000 ng/mL, respectively and 92.7% for IS.

#### 2.2.5. Stability

The results of stability test for GP205 were summarized in Table 3. The data demonstrated that GP205 in rat plasma was all stable for three freeze-thaw cycles, at least 4 h at the room temperature, 40 days under −80 °C and in a period of 24 h in the auto-sampler with accuracy in the range of −7.7%–−1.6%. The stability of GP205 in stock and working standard solutions were stable for at least 24 h at room temperature and 40 days at 4 °C with the accuracy of −0.3%–3.3%.

#### 2.2.6. Sample Dilution

To demonstrate the ability of diluting plasma samples containing GP205 at concentrations above the ULOQ, a set of plasma samples was prepared containing GP205 at concentrations of 8000 ng/mL and 20,000 ng/mL and stored at −80 °C overnight prior to analysis. After thawing at room temperature, the spiked samples were diluted with blank rat plasma to generate the final concentration of 4000 ng/mL. Then the samples were prepared and analyzed. The results of sample dilution were shown in Table 4, which demonstrated that diluting high concentration samples with blank plasma could not affect the accuracy and precision of the assay.

**Table 3:** Stability of GP205 in SD rat plasma (*n* = 3).

Sample Condition	Nominal Conc. (ng/mL)	Measured Conc. (ng/mL)	Accuracy (%)
Bench top stability ^a^	5	4.84	−3.3
	100	98.39	−1.6
	4000	3884.71	−2.9
Auto-sampler stability ^b^	5	4.79	−4.2
	100	96.13	−3.9
	4000	3904.44	−2.4
Freeze-thaw stability ^c^	5	4.73	−5.5
	100	94.90	−5.1
	4000	3925.86	−1.9
Long time stability ^d^	5	4.61	−7.7
	100	92.84	−7.2
	4000	3812.43	−4.7
Room temperature stabilityfor stock solution ^e^	5	5.09	1.8
	100	103.26	3.3
	4000	3987.91	−0.3
At 4 °C stability for stock solution ^f^	5	5.14	2.8
	100	100.63	0.6
	4000	3992.75	−0.2

^a^ Exposed at ambient temperature (25 °C) for 4 h. ^b^ Kept at auto-sampler (25 °C) for 24 h. ^c^ After three freeze-thaw cycles. ^d^ Stored at −80 °C for 40 days. ^e^ Stored at 25 °C for 24 h. ^f^ Stored at 4 °C for 40 days.

**Table 4:** Summary of dilution effects of GP205 in SD rat plasma (*n* = 3).

Dilution Factor	Assayed Concentration (ng/mL)	Reported Concentration (ng/mL)
2	4426.87	8853.74
	4234.72	8469.44
	4347.85	8695.70
	Mean	8672.96
	RSD (%)	2.3
	Accuracy (%)	8.4
5	4365.77	21,828.85
	4039.57	20,197.85
	4104.06	20,520.30
	Mean	20,849.01
	RSD (%)	4.2
	Accuracy (%)	4.3

Nominal concentration: 8000, 20,000 ng/mL for GP205.

### 2.3. Pharmacokinetic Study

**Figure 4:**
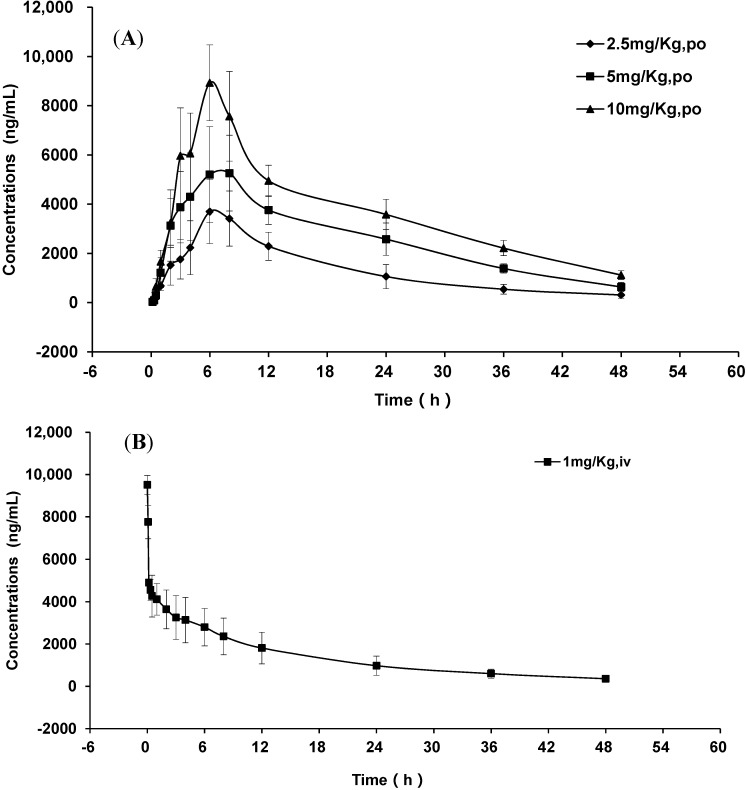
Mean plasma concentration-time profiles of GP205 (each point represents mean ± SD). (**A**) mean plasma concentration-time profiles after oral administration of GP205 at the doses of 2.5, 5, 10 mg/kg in SD rats; (**B**) mean plasma concentration-time profiles after intravenous administration of GP205 at the doses of 1 mg/kg in SD rats.

The method developed above was applied to a pharmacokinetic study of GP205 to SD rats after single oral doses at 2.5, 5 and 10 mg/kg and an intravenous injection dose at 1 mg/kg. A representative chromatogram from a 2.5 mg/kg dosage plasma sample is shown in Figure 3E. The mean plasma concentration-time profiles of GP205 of different doses are shown in Figure 4.

The concentration-time data were analyzed by non-compartmental method using the Bioavailability Program Package (BAPP, Version 3.1, Center of Drug Metabolism and Pharmacokinetics, China Pharmaceutical University, Nanjing, China) and the pharmacokinetic parameters are shown in Table 5. In our study, the plasma drug concentration reached the maximum point at about 6 h after oral administration and it did not change significantly with administration dosages. The plasma exposure parameters of the maximum concentration (*C*_max_) and area under curve (AUC) were both high and linear with the oral administration dosage ranging from 2.5 to 10 mg/kg. The linear regression of *C*_max_ = 663.06 Dose + 2948.33 (*r*^2^ = 0.91) and AUC_0-48_ = 14348.15 Dose + 37279.43 (*r*^2^ = 0.89). After single oral administration of GP205 at the doses of 2.5, 5, 10 mg/kg to the SD rat, the plasma concentration of GP205 decreased gradually with an elimination half time (t_1/2_) of approximately 12.38 h~17.21 h, which was longer than the t_1/2_ (about 6 h) of MK-1220.

**Table 5:** Mean pharmacokinetic parameters after oral and intravenous administration of GP205 in 24 SD rats.

Pharmacokinetic Parameters	GP205		
Oral	2.5 mg/Kg	5 mg/Kg	10 mg/Kg
*T*_max_ (h)	6.30 ± 0.80	6.30 ± 1.50	6.30 ± 0.80
*C*_max_ (ng/mL)	4260.32 ± 400.35	6782.15 ± 749.52	9406.12 ± 582.99
*t*_1/2_ (h)	12.38 ± 1.51	13.91 ± 1.48	17.21 ± 2.73
MRT (h)	19.35 ± 3.22	22.17 ± 2.18	25.55 ± 3.48
AUC_0-τ_ (ng·h/mL)	64,342.95 ± 13,770.51	122,230.48 ± 9225.20	176,357.52 ± 16,331.60
AUC_0-∞_ (ng·h/mL)	70,136.75 ± 16,623.71	135,312.81 ± 13,439.93	204,693.19 ± 15,949.70
IV	1 mg/Kg		
*C*_0_ (ng/mL)	9511.56 ± 442.31		
*t*_1/2_ (h)	16.26 ± 2.99		
MRT (h)	20.76 ± 3.41		
Cl(L/h/Kg)	0.02 ± 0.01		
V_D_(L)	0.31 ± 0.12		
AUC_0-τ_ (ng·h/mL)	66,640.04 ± 21,721.23		
AUC_0-∞_ (ng·h/mL)	75,210.88 ± 23,751.74		

The *C*_max_ and AUC of GP205 were about 2 fold and 11 fold higher than those of MK-1220, respectively [50]. The existing NS3/4A inhibitors are cleared from plasma relatively rapidly after oral administration and should be dosed twice or thrice daily to maintain sufficient drug plasma concentrations. GP205 has the advantage of slow elimination, and could achieve significant plasma levels following oral dosing in SD rat, indicating potential for the treatment of HCV. The oral bioavailability of GP205 in rat was 34%, which was moderately less than that of MK-1220 (37%). The introduction of the *tert*-butoxycarbonylamino moiety to the P2-P4 ring of GP205 might change the lipophilicity of the compound and affect its absorption, which resulted in the different absolute bioavailability. The approved NS3/4A inhibitors boceprevir and telaprevir are advised to be administrated with food in order to enhance the exposure relative to the fasting state [56]. Further preclinical research on GP205 could focus on the oral bioavailability with or without food, tissue distribution, especially liver exposure, metabolism and excretion based on the pharmacokinetic parameters obtained from this study.

## 3. Experimental Section

### 3.1. Chemicals and Reagents

GP205 (potassium *tert*-butyl N-[(3*R*,5*S*,8*S*,11*S*,15*E*)-8-cyclohexyl-5-{[(1*R*,2*S*)-1-[(cyclo-propanesulfonyl) carbamoyl]-2-ethenylcyclopropyl] carbamoyl}-18-methoxy-7,10-dioxo-2,13-dioxa-6,9,23-triazatetracyclo[15.6.2.13,6.020,24]hexacosa-1(23),15,17(25),18,20(24),21-hexaen-11-yl] carbamate monohydrate, 99.49% purity, C_43_H_57_KN_6_O_12_S, MW 921.11) was provided by Ginkgo Pharma Co. Ltd. (Suzhou, China). Paclitaxel (IS) was purchased from National Institutes for Food and Drug Control (Beijing, China). Chromatographic grade formic acid, methanol and methyl *tert*-butyl ether was purchased from Tedia (Fairfield, CA, USA), Merck (Darmstadt, Germany) and J.T. Baker (Phillipsburg, NJ, USA), respectively. Deionized water (18.2 MΩ/cm) was generated in-house using a Milli-Q system from Millipore (Bedford, MA, USA).

### 3.2. LC-MS/MS Instrumentation

A Varian HPLC-MS/MS system (Palo Alto, CA, USA) consisting of a Pro-Star 430 auto-sampler, two Pro-Star 210 pumps and a 1200 L triple quadrupole mass spectrometer equipped with an electro-spray ionization source was used. Varian MS workstation version 6.8 software was used for data acquisition and processing.

### 3.3. Liquid Chromatographic Conditions

The chromatographic separation was performed on an Agilent ZORBAX SB-C18 (100 × 3.0 mm, 3.5 μm) column at a column temperature of 25 °C. The auto-sampler temperature was kept at an ambient temperature of 25 °C. The mobile phase comprised methanol–water–formic acid (95:5:0.1, v/v/v) and was pumped at a flow rate of 0.3 mL/min. The injection volume was 10 μL and the analysis time was 4 min per sample.

### 3.4. Mass Spectrometer Conditions

The mass spectrometer was operated in positive ionization mode. The electro-spray capillary voltage was set to 85 V. Nitrogen was used as a drying gas for solvent evaporation. The drying gas temperature was kept at 200 °C. Protonated analyte molecules were subjected to collision induced dissociation using argon as the collision gas to yield product ions for the analyte and IS. The collision energy was −35.0 eV and −21.5 eV for GP205 and IS, respectively. The scan time was 1s and the detector multiplier voltage was set to 1800 V. GP205 and the IS paclitaxel were simultaneously monitored by MRM modes. The MRM transitions for GP205 and IS were *m/z* 887.5→787.4 and *m/z* 876.3→307.8, respectively.

### 3.5. Preparation of Standard and Quality Control (QC) Samples

Stock solutions of GP205 were prepared by dissolving an accurately weighed amount of reference standard in methanol to yield a final concentration of 1 mg/mL. The solutions were sonicated for 5 min to ensure complete dissolution. Then the solutions were allowed to equilibrate to room temperature and diluted to volume with methanol. Working standard solutions of GP205 in the concentration range of 20–50,000 ng/mL were prepared from individual aliquots of 1 mg/mL solution using methanol–water (95:5, v/v) as the diluent. The stock standard solution of IS was prepared by dissolving appropriate amount of paclitaxel in methanol at the concentration of 1 mg/mL. A 1 μg/mL IS working solution was obtained by diluting the stock solution of paclitaxel with methanol–water (95:5, v/v). All the solutions were stored at 4 °C and brought to room temperature before use. Calibration standards were prepared daily by spiking 5 μL of the appropriate standard solutions to 45 μL of the blank SD rat plasma. Plasma concentrations were 2, 5, 10, 50, 100, 200, 500, 2000 and 5000 ng/mL for GP205. QC samples, which were used in the validation and during the pharmacokinetics study, were prepared from different sources by independent dilution at three levels (5, 100, 4000 ng/mL) for GP205. QC samples were aliquoted into 1.5 mL non-sterile Eppendorf tubes and stored at −80 °C until analysis.

### 3.6. Extraction Procedure

In order to increase sample throughput, the LLE in 96-well format plate was used, which resulted in shorter sample preparation time. Prior to analysis, the plasma samples of SD rats were taken out from −80 °C freezer and kept at room temperature for thawing. The samples were vortexed adequately using a vortex mixer before pipetting. Samples were prepared using LLE in 96-well deep format plate (2 mL, Corning Life Sciences—Axygen Inc, Union City, CA USA). An automatic multichannel pipette (INTEGRA Biosciences AG, Zizers, Switzerland) was used for liquid transfer steps. Aliquots of 25 μL plasma were transferred into 96-well deep format plate. To a 25 μL aliquot of plasma sample, 2.5 μL of IS working solution (1 μg/mL) was added and vortexed to mix. The mixed sample was then extracted with 250 μL methyl tert-butyl ether by vortex-mixing for 3 min in a platform shaker. After centrifugation at 8000 rpm for 10 min, 200 μL of the upper organic layer was transferred to 96-well microplate (200 μL). Extracts were concentrated to dryness by automated concentration evaporation system at 40 °C under gentle stream of nitrogen (15 psi, 1.03 × 105 Pa) (Turbo Vap 96, Biotage AB, Uppsala, Sweden) for 10 min. The residue was reconstituted with 50 μL of methanol–water (95:5, v/v). And then it was vortexed adequately for 3 min and centrifuged at 12,000 rpm for 3 min. A 10 μL aliquot of the supernatant was injected into the LC-MS/MS system for analysis.

### 3.7. Pharmacokinetic Study in Rats

Twenty four SD rats (12 males and 12 females), weighing 210 ± 8 g (mean ± SD) were fed with certified standard diet and tap water ad libitum. Temperature and humidity were regulated at 21–23 °C and 30%–60%, respectively. A light/dark cycle of 12 h on/12 h off was established. After 1 week of acclimatization, they were randomly divided into 4 groups (3 males and 3 females for each group). After fasted overnight, three groups of them received oral administration of GP205 at three dosages 2.5 mg/kg, 5 mg/kg, 10 mg/kg dissolved in mixed solvent (5% DMSO, 40% PEG-400, 55% physiological saline), respectively. The other group of them received an intravenous injection of GP205 at 1 mg/kg. Animal had free access to water and food 4 h after drug administration. Blood samples (100 μL) were collected into heparinized tubes before administration and at 0.083, 0.17, 0.33, 0.5, 1, 2, 3, 4, 6, 8, 12, 24, 36, 48 h after oral administration or before administration and at 0, 0.083, 0.17, 0.33, 0.5, 1, 2, 3, 4, 6, 8, 12, 24, 36 , 48 h after intravenous administration. The plasma was separated from heparinized blood by centrifugation at 3500 rpm for 10 min and stored at −80 °C prior to analysis.

## 4. Conclusions

In this study, a sensitive, rapid and reliable LC-MS/MS method for the determination of GP205 in rat plasma has been developed and validated for the first time. This method also produces accurate and precise measurements of GP205 plasma concentration with a small plasma volume of 25 μL and in a short run time of 4 min, which satisfy the requirements of high sensitivity, specificity and rapid sample throughput. The results for parameter validation such as recovery, matrix effect and stability are within the acceptable limits. The linearity is good over the linear range of 2–5000 ng/mL to investigate the GP205 in SD rat plasma. Unknown samples concentrations exceeding the range were diluted to one second or fifth with control blank plasma and re-assayed. The analytes were found to be stable in SD rat plasma for 40 days when stored at −80 °C. The pharmacokinetic behavior of GP205 was more inclined to show linear pharmacokinetics in the oral administration dosage ranging from 2.5 to 10 mg/kg. The absolute bioavailability of GP205 in rat was estimated as 34%. The long half lifetime (12.38 h–17.21 h) of GP205 implies the slow elimination of GP205 with sufficient plasma exposure for a longer time. GP205 thus emerges as a suitable candidate for further development. Further studies are needed to investigate the tissue distribution, especially liver exposure of GP205 to make sure the target tissue as well as the oral bioavailability with or without food, metabolism, and excretion of GP205 in preclinical studies.
